# Effect of an Integrated Physiotherapy Protocol on Knee Osteoarthritis Patients: A Preliminary Study

**DOI:** 10.3390/healthcare11040564

**Published:** 2023-02-14

**Authors:** Sohrab Ahmad Khan, Prithvi Parasher, Mairaj Ahmed Ansari, Suhel Parvez, Noor Fatima, Iqbal Alam

**Affiliations:** 1 Department of Physiotherapy, Jamia Hamdard, New Delhi 110062, India; 2 Department of Biotechnology, SCLS, Jamia Hamdard, New Delhi 110062, India; 3 Department of Toxicology, SCLS, Jamia Hamdard, New Delhi 110062, India; 4 Department of Molecular Medicine, SIST, Jamia Hamdard, New Delhi 110062, India; 5 Department of Physiology, Hamdard Institute of Medical Sciences and Research, New Delhi 110062, India

**Keywords:** exercise therapy, manual therapy, physiotherapy, knee osteoarthritis, pain, balance, functional performance, disability

## Abstract

Background: Exercise therapy can potentially relieve symptoms and improve functional status of the knee osteoarthritis population. Despite the proved practical benefits, there is no standard, comprehensive physiotherapeutic protocol available targeting the physical and physiological impairment cluster associated with disease. Osteoarthritis is a whole joint disease, affecting joint cartilage, ligaments, menisci and joint associated muscles, from variable pathophysiological processes. Hence, there is a need to develop a physiotherapy protocol to address the multi-structural physical, physiological and functional impairments associated with the disease. Objective: The objective of the present study is to evaluate the efficacy of designed, therapist supervised, patient education, progressive resistance exercises, passive stretching exercises, soft tissue manipulation, muscle energy technique, Maitland mobilization, aerobic exercise, and neuromuscular training physiotherapy protocol on pain, disability, balance, and physical functional performance in knee osteoarthritis patients. Methodology: The preliminary study was conducted on a (*n* = 60) sample of convenience. The samples were randomly allocated into two study groups, intervention, and control group. The control group was advised on a basic home program. On the other hand, the treatment of the intervention group was designed with a therapist supervised Physiotherapy Protocol. The outcome variables studied were the Visual Analogue Scale, Modified WOMAC Scale, Timed Up and Go Test, Functional Reach Test, 40 m Fast Paced Walk Test, Stair Climb Test, 30 s Chair Stand Test. Results: The results of the study revealed a significant improvement among most of the studied outcome measures in the intervention group, hence the designed supervised physiotherapy protocol was found effective in relieving multiple physiological impairments associated with this whole joint disease.

## 1. Introduction

Osteoarthritis, is the most common articular disease, predicted to be the fourth leading cause of disability worldwide [1]. In the majority, the disease accounts for knees, hips, hands, facets, and feet joint impairments, however, of the total disease burden, the knee joint accounts for 83% [2]. Osteoarthritis of the knee joint significantly affects quality of life, morbidity, mortality and is reported to negatively impact the economic status of the affected population [3]. In 2020, a global prevalence of 16% was reported in the population aged fifteen years and over, and 22.9% of the population (654.1 million people) aged forty years and over [4]. In India the reported prevalence of osteoarthritis of the knee joint among the population over fifty years old, was 17–61% [5]. In the population with osteoarthritis of the knee joint, pain and impaired function are the main highlighted problems that cause mobility impairments, that renders difficulty in carrying out daily life activities, resulting in poor quality of life [6]. The sufferers also presented impaired proprioception, that could potentially hamper individual’s postural stability and increase fall risk [7,8,9,10].

Currently, we do not have any therapy that could address all of the pathological structures involved [11]. There are some management strategies available which target different phenotypic components of the disease. The limited utility of some of the treatment options, comparative to the disadvantages and complications associated, are a matter of concern. Gene therapy, a novel approach, is still under evaluation. [11]. Additionally, joint replacement surgeries are quite cumbersome procedures, expensive for patients’, and associated with various complications as well as requiring tertiary care services. India, being a developing country, has tertiary care that is mostly out of the reach of common people. The recommendations given by the European League Against Rheumatism (EULAR), suggested more focused research work to achieve promising therapies or strategies to combat knee osteoarthritis. Therefore, special attention was drawn towards non cartilaginous tissues, intra articular structural interactions, early disease progression, and pathogenesis of disease associated pain to achieve effective treatment strategies [12].

There are various studies that suggested many strategies, but none of the strategies have a holistic approach. In one of the studies, the exercise therapy alone potentially relieved symptoms and improved functional status of the knee osteoarthritis population [13,14]. The National Institute for Health and Care Excellence (NICE) suggested aerobic fitness and strengthening exercises [13,15]. The Osteoarthritis Research Society International (OARSI) suggested two types of land-based structured exercise programs i.e., balance or neuromuscular training and/or strengthening and/or cardio or secondly mind-body exercises [13,16]. The American College of Rheumatology (ACR) suggested aquatic, aerobic and/or resistance exercises [13,17]. The Ottawa panel suggested strengthening exercises with or without other types of exercises, aerobic exercises with or without strengthening exercises, and mind-body exercises [13,18,19,20]. For managing knee joints, the EULAR suggested speeding up activity and exercise in general [13,21].

Despite the proven practical benefits, there is no standard, comprehensive physiotherapy protocol available targeting the physical and physiological impairment cluster associated with the disease. Since, osteoarthritis is a whole joint disease affecting joint cartilage, ligaments, menisci and muscles, from variable pathophysiological processes, it requires a holistic approach to address all components of osteoarthritis [22]. Although a number of protocols are available for the foreign population, the Indian population are unable to be treated under the same umbrella. The Indian population as well as interacting environmental characteristics are not the same as the foreign population, consequently the protocols used by them cannot be implied to treat the Indian population. The Indian population is highly tolerant, on top of that, the physical environment including the residential and working environment is challenging. The tolerance of the Indian population can be reflected in their ability to ignore pain and their delayed treatment seeking behavior.

The delay in the disease management causes advancement in the pathology which renders progressive involvement of various articular and peri-articular structures, causing multiple impairments like pain, balance impairment, functional limitation and disability. Hence, there is a need of an integrated physiotherapy protocol for the Indian population, to address the multistructural involvement of the diseased knee joint and help to manage the pain, impaired balance, functional limitation and disability.

The current study aims to provide an integrated physiotherapy protocol for Indian knee osteoarthritis patients that targets the provision of a holistic approach to manage physical and physiological impairments associated with the different structures of the involved knee joint. The approach we are reporting in this study is not only efficient in combatting osteoarthritis but is also cost effective, and would be easily available for the population.

## 2. Materials and Methods

### 2.1. Study Design

The preliminary study with analysis by protocol, consisted of a sample of convenience of sixty participants who were randomly allocated into two groups, the intervention group (*n* = 30) and control group (*n* = 30). To randomly allocate participants to the intervention and control group; well-shuffled, identical chits having names of all sixty participants were kept in a jar. An individual, who was not a part of the study, was asked to draw one chit at a time from the jar and put that into one of two bowls, each bowl, respectively, marked as the Intervention or Control Group. The two study groups were kept homogeneous. The study was approved by the Institutional Review Board, Jamia Hamdard, New Delhi, India. Written informed consent was taken from all participant patients before their participation in the study.

### 2.2. Participants

The patient sample pool was selected from the knee osteoarthritic patients referred to the Rehabilitation Center, Jamia Hamdard from Hakeem Abdul Hameed Centenary Hospital, HIMSR, Jamia Hamdard. The included age group was forty-five to sixty years. Clinically, diagnostic confirmation of knee osteoarthritis was performed according to American College of Rheumatology criteria. Patients having a history of ligament and/or meniscal injury at the level of the knee joint; history of fracture in the last six months; history of cancer; history of other systemic diseases; history of metabolic diseases; history of infectious diseases; history of neuromuscular diseases; patients who had undergone knee joint replacement surgery or had been injected intra-articularly with corticosteroid at the level of the knee joint, within a period of six months; pregnant or lactating women; known cases of ankylosing spondylitis, rheumatoid arthritis, osteomalacia, Paget’s disease. Subjects having body mass index greater than thirty-five; subjects having osteoarthritis higher than grade-II; patients involved in or about to participate in any other structured lower limb strengthening program were all excluded.

### 2.3. Intervention

Individuals in the control group were given handouts of a basic home program containing knee isometric exercises with self-stretching exercises for quadriceps femoris, hamstrings, gastro-soleus and precautionary advice of Dos and Don’ts regarding knee osteoarthritis. The intervention group was administered with a designed therapist supervised PEPSMAN physiotherapy protocol, consisting of Patient Education, Progressive resistance exercise, passive Stretching exercises and Soft Tissue Manipulation (STM), Muscle Energy Technique (MET) and Maitland mobilization, aerobic exercise, and neuromuscular training, three sessions per week for four weeks.

The patients’ education session was conducted once a week during the entire study period, hence a total of four education sessions were conducted. Each education session was planned accordingly, so that the next session was a progressive series of the previous one. Each individualized session was for a one-and-a-half-hour period. The sessions were conducted in a well-lit, ventilated, comfortable environment without interference of any external disturbances. The first session was aimed at providing elaborated knowledge of the disease to the patient, understanding the patients’ beliefs related to the disease, and his/her condition. The first session consisted of a detailed explanation of what knee osteoarthritis is, its etiological factors, related articular and periarticular changes, need for treatment, importance of precautions, importance of physiotherapy and role of our protocol; everything was discussed in layman terms. The second session was aimed at motivating patients, correcting his/her misbeliefs, negative experiences, and false expectations.

In the third session, the patient was again motivated by discussing the further achievements, and patient experienced improvements. The learnings from the second session were further intensified by discussing what patients’ false expectations were and correct information was given to the patients and the problems were tackled accordingly. The last session comprised of bushing up on the learnings of all the previous three sessions. The aim of this session was to impart knowledge regarding future aspects and practices like, maintenance of precautions, practical Dos and Don’ts, importance of regularly practicing prescribed physical activity, dangerous ill effects of treatment avoidance and health negligence, importance of healthy weight management, strategies for maintaining healthy lifestyles, ergonomics, and the importance of regular follow-up.

The elaborative explanation of the intervention program is mentioned in Table 1. During the study, none of the subjects undertook any chondroprotective-anti-inflammatory intra-articular agent or physiotherapy program other than the prescribed one.

### 2.4. Outcome Measure

Primary outcome measures assessed were, pain intensity, assessed through the Visual Analogue Scale (VAS), The visual analogue scale represent a straight-line describing the limits of pain, the two ends of the line reflect two extremes of pain, no pain at all and worst pain [33,34]. With respect to the subjective nature of the pain, the patient was asked to mark the perceived pain level on the scale [34] during pre-test and post-test examination. The difference between the two differently reported points on the scale by an individual at different points of time, reflected the real variation in pain magnitude [34,35,36].

The modified Western Ontario and McMaster University (WOMAC) Osteoarthritis Index was utilized to assess functional mobility, quality of life, and activities of daily living [37]. The index was administered to the participants and the participants were asked to report the index accordingly, during the pre-test and post-test evaluation periods.

The timed Up and Go test (TUG) was conducted during the pre-test and post-test period, to assess dynamic steady state balance of the participants [38]. Time taken by the subject to get up from the arm support-less chair, walking five meters at self-selected speed, returning, and occupying sitting a position again over the chair; was measured to serve this purpose.

The functional reach test (FRT) was also intended to assess dynamic steady state balance [38]. The participants were asked to stand initially with an outstretched arm with a fist hand, the initial position of the knuckle of the third metacarpal was marked, then he/she was asked to attain a final position of maximal forward reach by leaning forward from the initial position without changing the base of support. The distance between the initial and final position of the knuckle of the third metacarpal was measured to serve the assessment purpose.

The forty meter fast paced walk test (40 m FPWT), was administered at both the pre-test and post-test evaluation period with an intention to assess the paced walking ability over short distances, ability to change direction, and speed during a short distance walk [39]. The participants were asked to walk speedily and safely on a ten-meter pathway, the end of the pathway was marked by a cone around which participants must turn around and return to the starting point marked with another cone, the activity was repeated to cover a forty-meter walkway in one go, without running [39]. Time was recorded from initiation to completion of the walking activity to measure the speed of the walk [39].

The stair climb test (SCT), assesses the strength and balance of the lower body, it also focusses on the ability of the individual to ascend or descend stairs [39]. In the pre-test and post-test measurement period the participants were asked to ascend a flight of stairs of nine steps and eight-inch step height at each step, then turn and descend back to the ground, the activity was asked to be performed in a quick and safe way [39]. The timing was recorded from initial position (both feet on the ground) to final position (both the feet on the ground again) [39].

The thirty second chair stand test (30 s-CST), was administered to assess the dynamic balance and strength of the lower body, it also emphasizes the individual’s sit to stand activity [39]. For assessment purposes the participants were asked to cross his/her arms over their chest, in a sitting position on a seventeen-inch high (seat height) chair with knees flexed at 90 degrees and feet resting on the ground at shoulder width apart [39]. The participants were asked to stand up and sit down repeatedly for thirty seconds, the number of repetitions made were recorded [39]. The measurement was carried out in the pre-test and post-test period [39].

### 2.5. Statistical Analyses

The analysis of the data was performed using SPSS Software Version 19 (SPSS Statistics: IBM Corporative, Chicago, IL, USA). To test the normality of the data, the Shapiro–Wilk Test was utilized. After satisfying the ANCOVA (analysis of covariance) assumptions using Levene’s Test of Equality of Error Variances (*p* > 0.05), the ANCOVA analysis was performed (Confidence Interval: 95%). Data was considered statistically significant if *p* < 0.05.

## 3. Results

**Figure 1:**
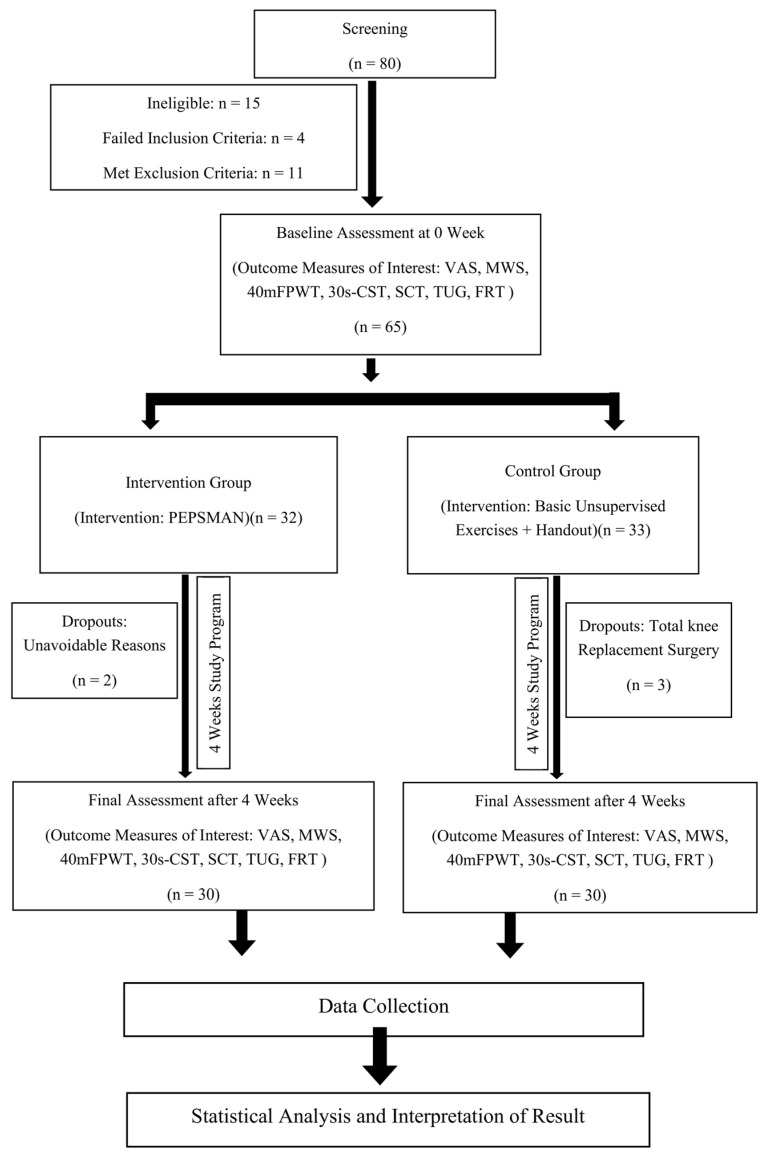
Flowchart of the participants and the study program.

Out of 65 subjects recruited in the preliminary study, three patients from the control group dropped out for a total knee replacement procedure during the study, the intervention group experienced two dropouts due to unavoidable reasons, hence a total of 30 subjects in each control and intervention group completed the study program. The characteristics of the subjects are described in Table 2. The flow of the participants during the study is represented in Figure 1.

**Figure 2:**
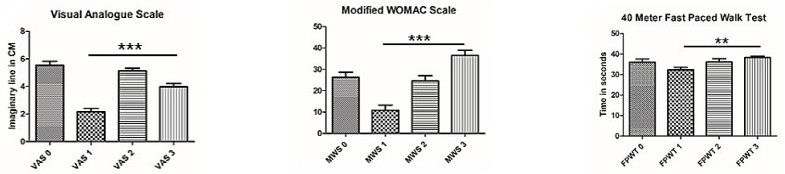

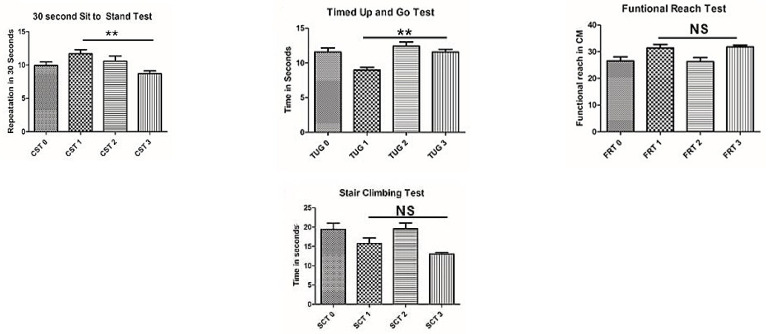
The mean data is adjusted for health, gender, and BMI. Data from thirty patients from each group have been analyzed and presented here in the bar graph with mean values and standard deviation. 0: Intervention group Pre; 1: Intervention group post treatment; 2: Control group pretreatment; 3: Control group post treatment. The *p* < 0.01 (**) and *p* < 0.001 (***) is denoted in the graphs, NS = Non significant.

The overall significance for the chosen factor i.e., PEPSMAN, in the test of between-subject effects and pairwise comparison was found to be statistically significant. Table 3, Table 4 and Table 5 and Figure 2 demonstrates statistical superiority of the independent variable through ANCOVA statistics, hence the administered intervention protocol, consisting of a combination of exercise therapy and manual therapy is found significantly effective in the management of knee osteoarthritis patients, however in case of the stair climb test and functional reach test, the data showed non-significant results. Additionally, the data showed evidence for aging as a factor impacting the slow response of the administered protocol.

## 4. Discussion

The purpose of conducting a preliminary study is to determine the medicinal potential of the designed therapist supervised PEPSMAN physiotherapy protocol in an Indian knee osteoarthritis population to relieve pain; improve static balance, dynamic balance, physical functional performance and hence, disability. Patient education is evidenced to have an important role in progressive diseases like osteoarthritis where the sufferers started surviving in a restricted environment that leads to poor social interactions [13,40,41,42]. Disturbed social health could potentially contribute to disturbances in mental and physical health. Recent studies supported administration of proper patient education to facilitate better management of disease [13]. Exercise therapy is a well-studied and accepted therapeutic strategy to improve the knee osteoarthritic diseased state, however, the work done in exploring the therapeutic potential of manual therapy alone and in combination with exercise therapy for the disease is scarce.

An administered, designed, therapist supervised PEPSMAN physiotherapy protocol i.e., combination of patient education, exercise therapy and manual therapy was found to have a significant therapeutic role in improvement of static balance, dynamic balance, pain, physical functional performance, and disability in patients with knee osteoarthritis. The result is evident in intervention data of the samples obtained after four weeks. The intervention group presented a significantly promising impact of the protocol, however, aging emerged as a potential factor that could slow down the therapeutic effect of the administered intervention. Aging being a common contributing factor of osteoarthritis, is well known to progressively cause cell senescence, altered apoptosis, mitochondrial dysfunction, oxidative stress, and homeostatic dysfunction; resultantly, the connective tissues comprising joints turned stiff, fragile and dehydrated. The continuum of deleterious changes associated with aging might be the reason for a delayed positive improvement with the protocol in the advanced age group, compared to the younger ones.

The analysis with respect to functional reach test and stair climb test unfolds a non-significant result. The functional reach test is a measure of balance. Although the outcome measure of balance is widely utilized in cases of lower limb impairments, the balance is not unique to the function of the lower extremities, it is an integrated function of lower limb and trunk stabilizers. The administered program was only targeting lower extremities; however, the trunk region was spared, hence this could be the possible reason of demonstrating an insignificant result in comparison to the other outcome measures of interest. In the case of the stair climb test, the reason behind the insignificant result is unknown, however, it is a matter of future research, hence studies focusing on the same area will be highly appreciated.

Though pain relief was obtained in both groups, the comparative improvement in pain between the two groups was not that significant. Exercise therapy and manual therapy individually, proved efficient to contribute to the symptomatic relief of the disease. Knee osteoarthritis being a chronic condition involves a psychological component of pain. Subjective belief and satisfaction could be the possible reasons behind the presented non-significant difference between the two groups, tested at the end of the study.

Static and dynamic balance improved significantly in the intervention group in comparison to the control group. The physiotherapeutic exercises and therapy administered to the intervention group was designed to improve muscle strength, flexibility, conditioning and joint proprioception, the improvement of these parameters might have potentially resulted in the improvement in balance of respective samples.

Functional performance and disability were investigated in the study with an intention to improve clinical practices of concern, to achieve patients’ independence and better life quality. With the administered protocol the intervention group achieved a significant improvement compared to the pre-test scores and control group, the potential mechanism behind the improved scores streamlines with symptomatic relief, improved muscle conditioning, joint sense and balance that was achieved with exercise therapy and manual therapy.

The result of the preliminary study is consistent with the study of Deyle et al., 2000, which demonstrated the effectiveness of supervised exercise program in achieving functional improvement in the knee osteoarthritis population when provided in combination with manual therapy [43]. Ko et al., 2009, made a favorable statement in a study that dealt with a knee osteoarthritis population, when they reflected a comparatively higher efficacy of a combination regimen of manual therapy and resistive exercise, than a regimen consisting of resistance exercise alone, in terms of bringing improvement in proprioception, functional performance and muscle strength [44]. Abbott et al., 2015, also presented supportive evidence by concluding the improved effectiveness of exercise therapy, with the addition of manual therapy [26].

The result of Abbott et al. 2013, was found to be contradictory to that of the current study. Abbott et al., 2013, concluded that exercise therapy and manual therapy when given in combination, in a single protocol was not found to be efficacious in relieving symptoms and improving function in a knee or hip osteoarthritis population, though exercise therapy or manual therapy when administered in isolation was evidently effective [45]. In the study conducted by Abbott et al., 2013, a recommendation was made pointing towards an antagonistic effect of exercise therapy and manual therapy, quoting it as potential reason for the contradictory result [45]. The current study revolves around a constructive approach with a background belief of summating the individual benefit of the combination of the administered individual therapies.

### 4.1. Strength of the Study

Many studies have investigated the effect of exercise therapy in knee osteoarthritis populations, however dealing with manual therapy the availability of literature is quite limited, hence the current study intended to contribute to the literature evidencing the impact of manual therapy. The study faced the COVID-19 pandemic era, irrespective of the challenging circumstances the sample size was attempted to be kept as high as possible, to have significant results. During the conduction of the study, special care was maintained to achieve allocation concealment. An individual alien to the study, performed participant allocation and the individuals’ taking the measurements were blinded to the source group of subjects. Several outcome measures were included in the study for the purpose of evaluating the variation of dependent variables. The study provides a complete evidence-based protocol to be practiced in the Indian knee osteoarthritis population to improve the diseased state.

### 4.2. Limitations of the Study

The study was conducted during the COVID-19 pandemic period, the period imposed a greater limitation in terms of availability of the samples, laboratories, and rehabilitation center. The study faced dropouts and was unable to include follow up due to difficulty in contacting patients because of the pandemic restrictions. Latest outcome measure tools with advanced technology were not included in the study because of limited accessibility of the resources.

### 4.3. Recommendations for Future Studies

There is a great need for good evidence to support the efficacy of non-pharmacology based, safer and cost-effective interventions. Future studies with a similar approach should be conducted with more advanced measurement techniques on larger sample sizes.

## Figures and Tables

**Table 1:** Designed PEPSMAN physiotherapy protocol administered to the subjects of the intervention group.

S. No.	Exercise	Description	Sets/Repetitions/Hold Time	Inter-Repetition/Inter-Set Rest Time	Duration	Supporting Evidence
1.	Patient Education	Four Sessions	-	-	4 weeks	Piyakhachornrot, N. et al., 2011 [23]; Goff, A.J. et al., 2021 [24]
2.	Progressive Resistance Exercise (PRE)	With patient specific resistance, for: Knee extensors Knee flexors Hip extensors Hip flexors Hip abductors Hip external rotators Ankle dorsiflexors Ankle plantar flexors	3 sets of 10 repetitions with 10 s hold	2 s between repetitions and 30 s between sets	4 weeks	Khan, A.A. et al., 2018 [25]; Abbott, J.H. et al., 2015 [26]
3.	Stretching Exercise	Therapist performed passive stretching of: Hamstrings Quadriceps femoris Gastro-Soleus	Single set of 3 repetitions with 30 s hold	30 s between repetitions	4 weeks	Anwer et al., 2018 [27]; Abbott et al., 2015 [26]
	Soft Tissue Manipulation (STM)	Peripatellar connective tissue Quadriceps femoris Hamstrings Hip adductor Gastro-Soleus Fascia of thigh musculature Fascia of leg musculature	3 to 5 min	-	4 weeks	Abbott, J.H. et al., 2015 [26]; Jorge, R.T. et al., 2015 [28]
4.	Muscle Energy Technique (MET)	Post isometric relaxation technique was administered after application of 15 min of superficial moist heat to: Quadriceps femoris Hamstrings Hip abductors Ankle plantar flexors	Single set of 5 repetitions, isometric contraction for 5 s and passive stretch for 10 s.	30 s between repetitions	4 weeks	Sartoyo, S. et al., 2022 [29]; Khan, A.A. et al., 2018 [25]
	Maitland Mobilization	Patellofemoral joint Tibiofemoral joint Distal tibiofibular joint Talocrural joint Talocalcaneal joint Hip joint	Specific to patient’s needs	-	4 weeks	Abbott, J.H. et al., 2015 [26]; Rangey, P.S. et al., 2015 [30]
5.	Aerobic Exercise	Pedo-Cycling: At patient specific self-selected speed and seat height	3 sets of 50 repetitions	30 s between sets	4 weeks	Luan, L. et al., 2021 [31]; Abbott, J.H. et al., 2015 [26]
6.	Neuromuscular Training:	Frenkel Exercise	3 sets of 15 repetitions	2 s between repetitions and 30 s between sets	4 weeks	Rodica Trăistaru et al., 2020 [32]; Abbott et al., 2015 [26]
		Standing balance on unstable surface	3 sets of 3 repetitions with 60 s hold	120 s between repetitions as well as between sets		

The program was tailored according to the specific needs of the individual patient.

**Table 2:** Demographic and clinical characteristics of control and intervention group.

Variables	Control Group (*n* = 30)		Intervention Group (*n* = 30)	
	Mean/Frequency	SD	Mean/frequency	SD
Gender:				
Male (*n*)	20		14	
Female (*n*)	10		16	
Age (years)	51.5	(5.5)	51.5	(5.2)
Height (cm)	160.6	(9.2)	159.1	(9.0)
Weight (kg)	70.4	(13.5)	71.1	(13.1)
Body Mass Index	27.8	(7.3)	28.02	(4.0)
Grade of Osteoarthritis	1.8	(0.8)	1.9	(0.5)
Diabetes (*n*)	5		7	
Inflammatory arthritis (*n*)	1		2	
Thyroid (*n*)	4		7	

**Table 3:** Descriptive Statistics.

Outcome Measures	PEPSMAN	Mean	SD	N
Visual Analogue Scale	Inter_P	5.53	1.613	30
	Inter_Po	2.17	1.234	30
	Non_P	5.13	1.008	30
	Non_Po	3.97	1.351	30
	Total	4.20	1.850	120
Modified WOMAC Scale	Inter_P	25.80	13.664	30
	Inter_Po	10.43	9.662	30
	Non_P	25.10	13.737	30
	Non_Po	37.00	7.497	30
	Total	24.58	14.755	120
40 m Fast Paced Walk Test	Inter_P	36.03	8.704	30
	Inter_Po	32.30	6.993	30
	Non_P	36.07	8.952	30
	Non_Po	38.33	3.198	30
	Total	35.68	7.558	120
30 s Chair Stand Test	Inter_P	9.93	2.947	30
	Inter_Po	11.70	3.292	30
	Non_P	10.57	4.329	30
	Non_Po	8.70	2.307	30
	Total	10.23	3.436	120
Stair Climb Test	Inter_P	19.41	8.699	30
	Inter_Po	15.77	7.811	30
	Non_P	19.53	8.545	30
	Non_Po	13.00	2.181	30
	Total	16.93	7.731	120
Timed Up and Go Test	Inter_P	11.58	3.276	30
	Inter_Po	8.97	2.266	30
	Non_P	12.43	3.191	30
	Non_Po	11.57	2.144	30
	Total	11.14	3.029	120
Functional Reach Test	Inter_P	26.50	8.444	30
	Inter_Po	31.43	7.295	30
	Non_P	26.30	8.197	30
	Non_Po	31.80	3.727	30
	Total	29.01	7.549	120

Key-Inter_Po: Intervention Group Pre-test Scores, Inter_P: Intervention Group Post-test Scores, Non_Po: Control Group Pre-test Scores, Non_P: Control Group Post-test Scores.

**Table 4:** Test of Between Subject Effects.

Source	Dependent Variable	Type III Sum of Squares	df	Mean Square	F	Sig.	Partial Eta Squared	Noncent. Parameter	Observed Power ^h^
Corrected Model	VAS	207.423 ^a^	6	34.570	19.554	0.000	0.509	117.325	1.000
	MWS	11,329.510 ^b^	6	1888.252	14.635	0.000	0.437	87.810	1.000
	FPWT	683.526 ^c^	6	113.921	2.105	0.058	0.101	12.632	0.736
	CST	157.733 ^d^	6	26.289	2.382	0.033	0.112	14.291	0.797
	SCT	1811.865 ^e^	6	301.978	6.439	0.000	0.255	38.632	0.999
	TUG	225.334 ^f^	6	37.556	4.896	0.000	0.206	29.378	0.989
	FRT	1148.139 ^g^	6	191.357	3.839	0.002	0.169	23.033	0.959
Intercept	VAS	17.504	1	17.504	9.901	0.002	0.081	9.901	0.877
	MWS	223.236	1	223.236	1.730	0.191	0.015	1.730	0.257
	FPWT	709.776	1	709.776	13.117	0.000	0.104	13.117	0.949
	CST	58.305	1	58.305	5.283	0.023	0.045	5.283	0.625
	SCT	180.330	1	180.330	3.845	0.052	0.033	3.845	0.494
	TUG	51.358	1	51.358	6.696	0.011	0.056	6.696	0.728
	FRT	1306.551	1	1306.551	26.211	0.000	0.188	26.211	0.999
Gender	VAS	1.058	1	1.058	0.598	0.441	0.005	0.598	0.120
	MWS	614.148	1	614.148	4.760	0.031	0.040	4.760	0.581
	FPWT	44.959	1	44.959	0.831	0.364	0.007	0.831	0.148
	CST	2.996	1	2.996	0.271	0.603	0.002	0.271	0.081
	SCT	10.367	1	10.367	0.221	0.639	0.002	0.221	0.075
	TUG	9.515	1	9.515	1.240	0.268	0.011	1.240	0.197
	FRT	171.653	1	171.653	3.444	0.066	0.030	3.444	0.452
age_yrs	VAS	0.239	1	0.239	0.135	0.714	0.001	0.135	0.065
	MWS	35.305	1	35.305	0.274	0.602	0.002	0.274	0.081
	FPWT	27.623	1	27.623	0.511	0.476	0.004	0.511	0.109
	CST	11.767	1	11.767	1.066	0.304	0.009	1.066	0.176
	SCT	702.350	1	702.350	14.975	0.000	0.117	14.975	0.970
	TUG	7.076	1	7.076	0.922	0.339	0.008	0.922	0.159
	FRT	20.015	1	20.015	0.402	0.528	0.004	0.402	0.096
BMI	VAS	0.359	1	0.359	0.203	0.653	0.002	0.203	0.073
	MWS	180.450	1	180.450	1.399	0.239	0.012	1.399	0.216
	FPWT	92.138	1	92.138	1.703	0.195	0.015	1.703	0.253
	CST	0.839	1	0.839	0.076	0.783	0.001	0.076	0.059
	SCT	255.285	1	255.285	5.443	0.021	0.046	5.443	0.638
	TUG	14.093	1	14.093	1.837	0.178	0.016	1.837	0.269
	FRT	33.370	1	33.370	0.669	0.415	0.006	0.669	0.128
PEPSMAN	VAS	206.275	3	68.758	38.892	0.000	0.508	116.675	1.000
	MWS	9779.811	3	3259.937	25.266	0.000	0.401	75.799	1.000
	FPWT	510.427	3	170.142	3.144	0.028	0.077	9.433	0.717
	CST	144.201	3	48.067	4.355	0.006	0.104	13.065	0.860
	SCT	894.736	3	298.245	6.359	0.001	0.144	19.077	0.963
	TUG	188.461	3	62.820	8.190	0.000	0.179	24.570	0.991
	FRT	825.118	3	275.039	5.518	0.001	0.128	16.553	0.934

Key-^a^. R Squared = 0.509 (Adjusted R Squared = 0.483) ^b^. R Squared = 0.437 (Adjusted R Squared = 0.407) ^c^. R Squared = 0.101 (Adjusted R Squared = 0.053) ^d^. R Squared = 0.112 (Adjusted R Squared = 0.065) ^e^. R Squared = 0.255 (Adjusted R Squared = 0.215) ^f^. R Squared = 0.206 (Adjusted R Squared = 0.164) ^g^. R Squared = 0.169 (Adjusted R Squared = 0.125) ^h^. Computed using alpha = 0.05.

**Table 5:** Estimates and Pairwise significance comparison.

				95% Confidence Interval	
Dependent Variable	PEPSMAN	Mean	Standard Error	Lower Bound	Upper Bound
Visual Analogue Scale	Inter_P	5.512 ^a^	0.244	5.028	5.995
	Inter_Po	2.145 ^a^	0.244	1.661	2.629
	Non_P	5.155 ^a^	0.244	4.671	5.639
	Non_Po	3.988 ^a^	0.244	3.505	4.472
Modified WOMAC Scale	Inter_P	26.277 ^a^	2.086	22.145	30.410
	Inter_Po	10.911 ^a^	2.086	6.778	15.043
	Non_P	24.623 ^a^	2.086	20.490	28.755
	Non_Po	36.523 ^a^	2.086	32.390	40.655
40 m Fast Paced Walk Test	Inter_P	36.151 ^a^	1.351	33.475	38.828
	Inter_Po	32.418 ^a^	1.351	29.742	35.094
	Non_P	35.949 ^a^	1.351	33.272	38.625
	Non_Po	38.215 ^a^	1.351	35.539	40.891
30 s Chair Stand Test	Inter_P	9.969 ^a^	0.610	8.760	11.178
	Inter_Po	11.736 ^a^	0.610	10.527	12.944
	Non_P	10.531 ^a^	0.610	9.322	11.740
	Non_Po	8.664 ^a^	0.610	7.456	9.873
Stair Climb Test	Inter_P	19.444 ^a^	1.258	16.953	21.936
	Inter_Po	15.798 ^a^	1.258	13.306	18.289
	Non_P	19.502 ^a^	1.258	17.011	21.994
	Non_Po	12.969 ^a^	1.258	10.478	15.461
Timed Up and Go Test	Inter_P	11.632 ^a^	0.509	10.624	12.640
	Inter_Po	9.022 ^a^	0.509	8.014	10.030
	Non_P	12.378 ^a^	0.509	11.370	13.386
	Non_Po	11.511 ^a^	0.509	10.504	12.519
Functional Reach Test	Inter_P	26.776 ^a^	1.296	24.207	29.345
	Inter_Po	31.709 ^a^	1.296	29.141	34.278
	Non_P	26.024 ^a^	1.296	23.455	28.593
	Non_Po	31.524 ^a^	1.296	28.955	34.093

Key-Inter_Po: Intervention Group Pre-test Scores; Inter_P: Intervention Group Post-test Scores; Non_Po: Control Group Pre-test Scores; Non_P: Control Group Post-test Scores. ^a^. Covariates appearing in the model are evaluated at the following values: Gender = 0.43, age_yrs = 51.48, BMI = 28.00.

## Data Availability

The data supporting the results reported in this paper can be freely accessed on request from the corresponding author, kindly consider iqbalasc@yahoo.com for any required information regarding this research paper.
